# Cardiac inflammation and diastolic dysfunction in hypercholesterolemic rabbits

**DOI:** 10.1371/journal.pone.0220707

**Published:** 2019-08-08

**Authors:** Walid Nachar, Nolwenn Merlet, Foued Maafi, Yanfen Shi, Teodora Mihalache-Avram, Mélanie Mecteau, Marine Ferron, Eric Rhéaume, Jean-Claude Tardif

**Affiliations:** 1 Montreal Heart Institute, Montreal, QC, Canada; 2 Université de Montréal, Montreal, QC, Canada; Scuola Superiore Sant'Anna, ITALY

## Abstract

**Background:**

Left ventricular diastolic dysfunction (LVDD) is present in more than 50% of patients suffering from heart failure. LVDD animal models are limited and its underlying mechanisms remain largely unknown. Aortic valve stenosis (AVS) may cause LVDD, and we recently reported LVDD in an AVS rabbit model. Here we aimed to develop a rabbit model of LVDD without AVS.

**Methods:**

Rabbits were fed with a 0.5% cholesterol-enriched diet (n = 9) or normal diet (n = 8) until they developed LVDD defined by a value of the echocardiographic parameter E/Em ratio higher than the mean at baseline + 2SD. Rabbits were then fed a 0.2% cholesterol-enriched diet for 4 weeks (average total diet duration: 20 weeks). Detailed cardiac structure and function measurements were assessed by echocardiography at baseline, weeks 8, 12 and 14 to 20, when applicable. Histological analyses and RT-qPCR were performed on LV samples.

**Results:**

The hypercholesterolemic diet induced LVDD without systolic dysfunction or AVS, as shown by multiple echocardiographic parameters, including early filling mitral peak velocity and deceleration rate, Em/Am ratio and E/Em ratio (all p<0.05), and by increased cardiac mRNA expression of brain natriuretic peptide (*Bnp*). Cardiac expression of mRNA for *Nox2*, *Vcam1*, *Mmp12*, *Mmp12*/*Timp1*, *Il1b* and *Col1*/*Col3* ratios was also higher in these rabbits (p<0.05). In contrast, cardiac *Sod2* mRNA expression was reduced in hypercholesterolemic rabbits compared to controls.

**Conclusion:**

Rabbits fed with a cholesterol-enriched diet develop LVDD with preserved systolic function and evidence of cardiac inflammation and oxidative stress. This rabbit model may be used in future studies to test treatment strategies against LVDD.

## Introduction

More than 6 million people suffer from heart failure in North America [1]. Around 30 billion dollars are spent yearly for this disease in the US, which include the cost of hospitalizations, medications, and missed days of work [2]. Heart failure with preserved ejection fraction (HFpEF; typically considered as EF≥50%) is present in more than half of heart failure cases [3]. The evaluation of diastolic function has become an integral part of a full echocardiographic study and the approach is described in the guidelines of the American Society of Echocardiography and the European Association of Cardiovascular Imaging [4]. The main risk factors for heart failure and left ventricular (LV) diastolic dysfunction (LVDD) remain coronary heart disease, myocardial ischemia, hypertension, aortic valve stenosis (AVS) and diabetes [5]. Patients with LVDD are treated for the contributing causes but not for the pathology itself. Physiologically, LVDD is usually the result of impaired LV relaxation and/or increased LV chamber stiffness, which increase cardiac filling pressures. Cardiac fibrosis, LV hypertrophy, inflammation and oxidative stress are frequently reported in LVDD studies [6–8], but the underlying cellular and molecular mechanisms are still not well understood. Animal models of LVDD are not abundant and more representative ones are needed. We recently demonstrated that a rabbit model of mild AVS, obtained by a 16-week period of 0.5% cholesterol-enriched diet supplemented with vitamin D_2_ (50 000 IU.day^-1^), also developed LVDD [9,10]. In this model, the correlations observed between LVDD and AVS suggested that the latter may have been responsible in part for the development of LVDD, but the direct consequences of the cholesterol-enriched diet were not explored [10]. In the current study, we aimed to develop a simple and relevant rabbit lipid-mediated LVDD model, without the presence of AVS or any surgical or genetic modification. This experimental model would allow a more comprehensive understanding of the importance of hypercholesterolemia on LVDD development, without the influence of other comorbidities. Assessment of LVDD was performed by serial echocardiography complemented by specific cardiac mRNA gene expression analysis and immunohistochemistry.

## Materials and methods

### Animals

Animal care and procedures complied with the Canadian Council on Animal Care guidelines and were approved by the Montreal Heart Institute Research Center ethics committee for animal research. New-Zealand White rabbits (3.0 ± 0.1 kg, aged 12–13 weeks) were fed with a 0.5% cholesterol-enriched diet (Teklad Global Rabbit Diet 2030, *Harlan Laboratories*, Madison, WI, USA) (n = 9) for 20 weeks or until they developed LVDD defined by a value of the echocardiographic parameter E/Em ratio higher than the mean at baseline + 2SD. Rabbits were then fed for 4 weeks with a 0.2% cholesterol-enriched diet (Teklad Global Rabbit Diet 2030, *Harlan Laboratories*) to preserve animals from potential hepatic problems. The total period of diet was an average of 20 weeks. Control rabbits were fed a normal diet and matched for diet duration (n = 8). At the end of the protocol, blood samples were obtained through the ear marginal vein under acepromazine tranquilization (1 mg/kg, i.m.). Rabbits were then sacrificed by exsanguination under anaesthesia after cardiac arrest in diastole (2% lidocaine, 5–10 mL, i.v.). Then, the LV was removed, flushed with sterile saline, and stored for histological analyses (immersion-fixed in 10% buffered formalin at 4 °C for 24 h and embedded in paraffin) and molecular studies (snap frozen in liquid nitrogen and stored at -80°C).

#### Echocardiography

Examinations were carried out with a phased-array 10S (4.5~11.5 Mega Hertz) probe using a Vivid 7 Dimension system (*GE Healthcare Ultrasound*, Horten, Norway). Intra-muscular injections of ketamine (35 mg/kg) and midazolam (0.9 mg/kg) were used for sedation. Complete echocardiography-Doppler examinations were performed at baseline, weeks 8, 12, 14, 15, 16, 17, 18, 19 and 20 to assess LV morphology, LV systolic and diastolic functions and aortic valve function parameters. Procedures were done as previously described [10].

#### RNA extraction and cDNA synthesis

Left ventricular tissue (200 to 400 mg) was homogenized in Trizol buffer using “PowerGen125” homogenizer (*Fisher Scientific*, ON, Canada) for approximately 60 seconds. Homogenates were then aliquoted into 5 tubes and one of the aliquot was used to extract total RNA using Qiagen miRNeasy Kit (*Qiagen*, ON, Canada). The quality and quantity of total RNA was assessed using Agilent RNA 6000 Nano Kit for Bioanalyzer 2100 System (*Agilent Technologies*, Santa Clara, CA, USA). RNA integrity numbers (RIN) were higher than 8/10 for all samples. One (1) μg of total RNA was used to generate the first strand of complementary DNA (cDNA) using High Capacity cDNA Reverse Transcription Kit with RNase inhibitor (*Applied Biosystems*, CA, USA) in a final reaction volume of 20 μl according to manufacturer’s instructions.

#### mRNA quantification using real-time PCR

Quantitative RT-PCR experiments were performed using a Bio-Rad CFX384 thermal cycler. Final reaction volume was 10 μl and composed of 1X SyberGreen (GoTaq qPCR Master Mix, *Promega Corporation*, Madison, WI, USA or Perfecta SyberGreenFastMix, ROX, *Quanta BioSciences*, Gaithersburg, MD, USA), 0.4 μM of forward and reverse primer, and 2.16 ng of cDNA template. Initial denaturation was made at 95°C for 5 min. Amplification was then performed during 40 cycles of denaturation at 95°C for 15 sec and annealing/extension at 60°C for 1 min. At the end, a dissociation curve was produced to analyze and confirm the specificity of the amplification by observation of a single peak in the curve. Standard curves of five to seven points were produced for each gene to transform sample’s Cts to relative expression values. Amplification efficiencies were calculated according to Pfaffl’s method [11]. All samples were run in duplicate and the mean values were used for calculations. Relative quantities of the unknown samples were normalized against the normalization factor (NF) which was calculated from the geometric means of the expression of the best two reference genes (*Gapdh*, *Sdha* and *Hprt1*) as selected by geNorm. Primer sequences are presented in S1 Table.

#### Histology and immunohistochemistry staining

Ventricles were harvested following sacrifice and processed as previously described [9]. Ventricular transverse sections (8 μm) were stained with Masson’s trichrome to assess LV fibrosis. Macrophage content was detected by RAM-11 staining as previously described [10]. Aortic valves were opened longitudinally, and the three valvular cusps were separated. Left coronary cusps were immediately frozen in an embedding medium and stored at -80°C. The region of analysis (ROA) was composed of 1000 μm of the Valsalva sinus from the leaflet base and 500 μm of the leaflet from the leaflet base as previously described [12]. Haematoxylin-phloxin-safran (HPS) and von Kossa stained sections were prepared for plaque examination and tissue calcification, respectively and as previously described [10,12]. Pictures were taken at 4X (HPS and Von Kossa staining on left coronary cusp), 10X (for RAM-11 and Sirius Red staining on LV sections) or 20X magnification (for Masson’s trichrome staining on LV sections) using a computer-based digitizing image system using a light microscope (*Olympus BX41*, Richmond Hill, ON, Canada) connected to a digital video camera Q-Color3 (*Olympus*, Richmond Hill, ON, Canada) using Image Pro Plus version 9.2 (*Media Cybernetics*, Bethesda, MD, USA) for picture acquisition and analysis (5 pictures/ LV section).

#### Dihydroethidium (DHE) staining

Superoxide production, a marker of oxidative status, was examined by staining fresh tissue sections with DHE as previously described [6], and with DAPI (*Sigma-Aldrich*, #D9542) as counterstaining. Dihydroethidium is oxidized in the presence of superoxide and forms fluorescent ethidium, which was detected by fluorescence with a Zeiss LSM-710 inverted confocal laser scanning microscope equipped with 410/502 nm excitation/emission filters. As a negative control, sections were incubated with 300 U/mL of superoxide dismutase (SOD, *Sigma-Aldrich* # S5395) before staining with DHE; fluorescent signal was abolished in comparison with other samples. A 20X magnification with a 0.8 zoom-out was used for picture acquisition. The analysis was performed with Image Pro Plus version 9.2 (*Media Cybernetics*) on 4 pictures/ LV section. Fluorescence was quantified by counting the pixels in correspondent image fields and normalizing to DAPI staining.

#### Biochemistry

C-Reactive Protein (CRP) levels were measured by ELISA using a commercial kit specific to rabbits (GenWay Biotech, San Diego, CA; GWB-9BF960). The serum of rabbits at end of the protocol was diluted (1:500) and processed following manufacturer’s instructions.

Total and unesterified cholesterol were measured on serum samples by an OD method using a commercial kit (*Wako* #439–17501 and #435–35801, respectively). Esterified cholesterol levels were obtained by subtracting unesterified cholesterol from total cholesterol.

#### Statistical methods

Continuous variables are presented as mean ± standard error of the mean (SEM). LVDD parameters and LV hypertrophy parameters were analyzed using repeated measures analysis of variance (ANOVA) model including one factor for time, one factor for group and the group-by-time interaction. When the interaction was significant, comparison of groups at each time point followed. One-way analysis of variance (ANOVA) including one factor for diet was used to compare between groups (ND/HCD/HCD+Vit D) followed by individual comparison between each diet group for valve histomorphometry experiments. Biochemical, histological and molecular results obtained at the end of the protocol were compared between groups by a t-test using the Satterthwaite or Pooled method, where appropriate. Pearson and Spearman correlation were calculated between inflammatory genes and echocardiographic parameters. Statistical analyses were performed using SAS version 9.2 or higher (*SAS Institute Inc*., Cary, NC, USA). All analyses were conducted at the 0.05 significance level.

## Results

Rabbits were fed a 0.5% cholesterol-enriched diet until they developed LVDD defined by a value of the echocardiographic parameter E/Em ratio higher than the mean at baseline + 2SD. According to this cut-off value, 3 of the 9 hypercholesterolemic rabbits were diagnosed with LVDD after a 15-week diet period, 2 rabbits after 16 weeks and 1 rabbit after 20 weeks of diet. Three rabbits died before meeting this criterion (at weeks 14, 16 and 18, respectively).

The serum levels of total, unesterified (free) and esterified cholesterol at the end of the protocol were markedly elevated in rabbits exposed to the high cholesterol diet compared to controls (Table 1). In particular, total cholesterol was increased by 24.8 mmol/L compared to the normal diet group (p = 0.032), corresponding to a 38-fold increase. However, the ratio between free and esterified cholesterol was similar between rabbits exposed to both diets (p = 0.944).

**Table 1:** Total, free and esterified cholesterol measured in rabbits’ serum at the end of the study.

	Normal diet group	High cholesterol diet group	p-value
Total cholesterol (mmol/L)	0.66 ± 0.10	25.5 ± 4.7	0.003
Free cholesterol (mmol/L)	0.25 ± 0.06	9.7 ± 2.6	0.015
Esterified cholesterol (mmol/L)	0.40 ± 0.05	15.8 ± 2.9	0.003

### High cholesterol diet induces progressive diastolic dysfunction with preserved systolic function in rabbits

**Fig 1:**
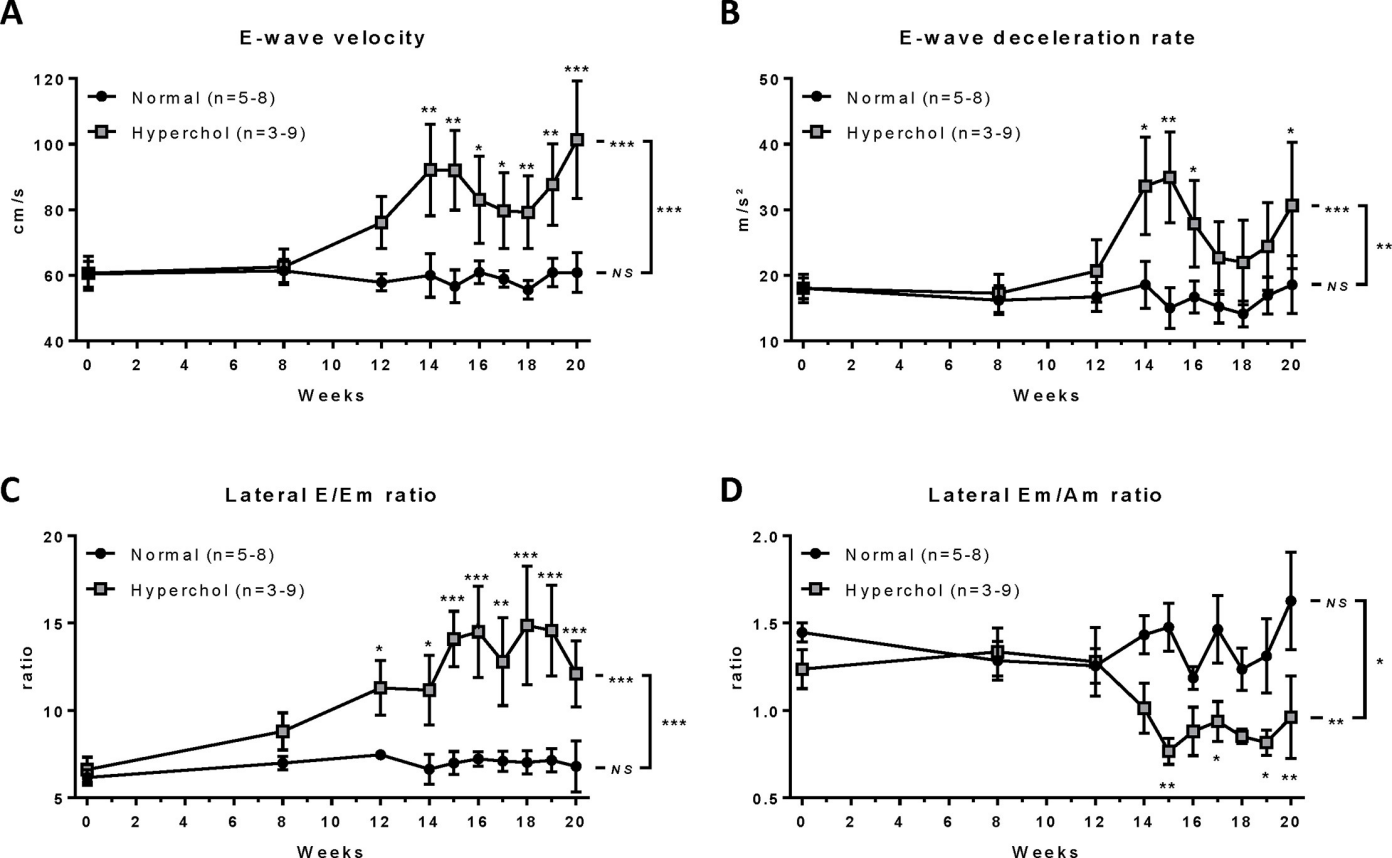
LVDD parameters assessed by pulsed wave and tissue Doppler echocardiography showing progression of the disease over time in rabbits fed with a normal diet (circles, n = 5–8) or a hypercholesterolemic diet (squares, n = 3–9). *p≤0.05, **p≤0.01, ***p≤0.001. Statistical analyses were performed to assess parameter’s change over time in each group, comparison between the change over time between groups and the differences between groups at each time points. Am: mitral annulus velocity during active atrial filling, E: peak velocity during early left ventricular filling, E-wave: early left ventricular filling, Em: mitral annulus velocity during early left ventricular filling, NS: non-significant.

**Fig 2:**
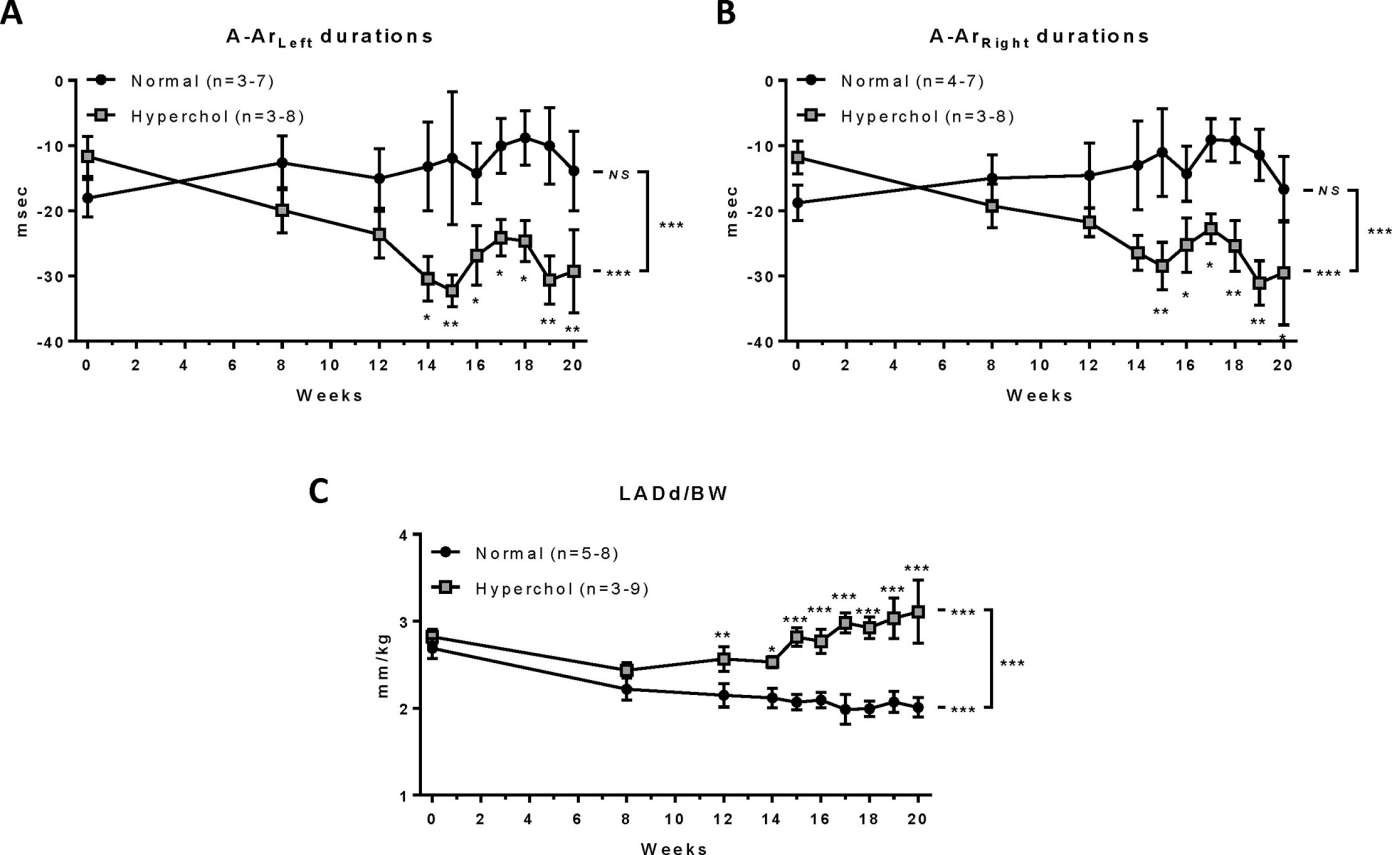
Pulsed wave Doppler at pulmonary venous levels. *p≤0.05, **p≤0.01, ***p≤0.001. Statistical analyses were performed to assess parameter’s change over time in each group, comparison between the change over time between groups and the differences between groups at each time points. A-Ar durations: mitral A-wave (active atrial filling) duration *minus* (left or right) pulmonary venous reversed atrial flow duration, LADd: smallest left atrium dimension at end cardiac diastole, BW: Body weight, NS: non-significant.

Progression of LVDD parameters was evaluated by echocardiography-Doppler at baseline (week 0) and at weeks 8, 12 and 14 to 20. From pulsed wave Doppler imaging of transmitral inflow, peak-velocity of early LV filling (E-wave) was significantly increased with hypercholesterolemic diet (p<0.001) but not in rabbits fed with a normal diet (p = 0.997), resulting in a significant over-time difference between the two groups (p<0.001; Fig 1A). Comparisons between groups at each time point indicate that this difference was significant as soon as 14 weeks of hypercholesterolemic diet. A similar pattern was observed for E wave deceleration rate (time*diet interaction: p = 0.003; Fig 1B). Based on tissue Doppler imaging of the lateral mitral annulus, the two indices of LV filling E/Em and Em/Am ratios were calculated. During the 20 weeks of high cholesterol diet, rabbits showed an increase of E/Em ratio (p<0.001; Fig 1C) and a decrease of Em/Am ratio (p = 0.002; Fig 1D), whereas no change was observed with the normal diet (p = 0.983 and p = 0.241, respectively), resulting in significant differences over time between groups (p<0.001 and p = 0.015, respectively). Also, the differences between the durations of mitral A-wave and pulmonary Ar-wave from both the left and right sides were significantly more negative (worsened) in hypercholesterolemic rabbits (p<0.001 *vs* baseline, both; Fig 2A and 2B) compared to normal rabbits (p = 0.528 and p = 0.336 *vs* baseline, respectively), resulting in significant differences over time between groups (p<0.001, both). Finally, left atrial diameter during diastole (LADd) was measured and indexed to rabbit body weight (LADd/BW). Whereas this index slightly but significantly decreased over time in the normal diet group (p<0.001), it markedly increased in hypercholesterolemic rabbits (p<0.001), resulting in a significant difference over time between groups (p<0.001; Fig 2C and S4 Fig). Taken together, these parameters are consistent with LVDD progression and increased left-sided filling pressures in hypercholesterolemic rabbits. While LVDD developed progressively over time, no significant changes were observed in LV systolic function parameters such as ejection fraction (p = 0.774) and fractional shortening (p = 0.787; S1 Fig), nor in heart rate (p = 0.486, S4 Fig).

### High cholesterol diet causes LV hypertrophy in rabbits

**Fig 3:**
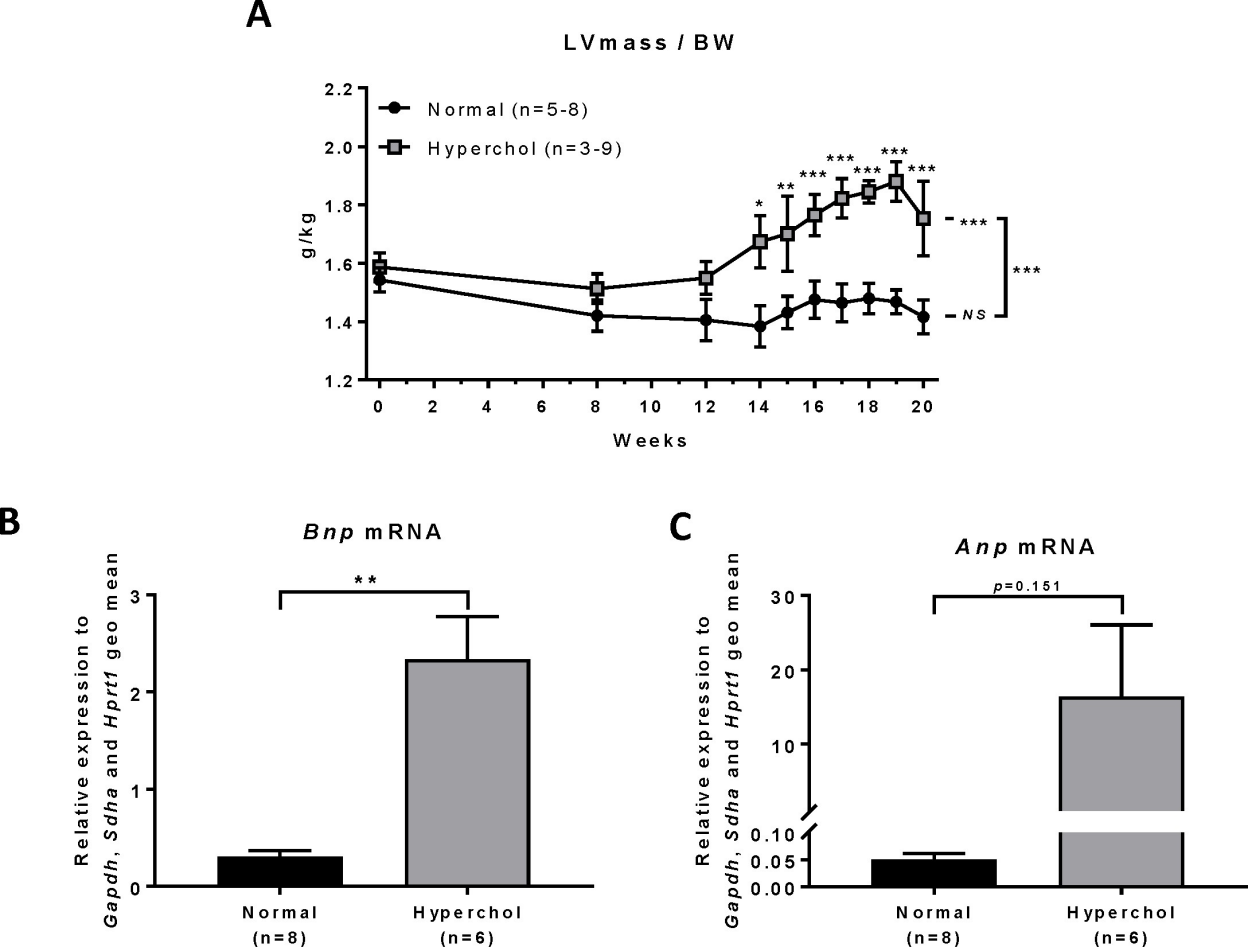
Cardiac hypertrophy as assessed by echocardiography and qPCR experiments. (A): Left ventricle (LV) mass normalized to body weight (BW) measured by echocardiography. (B) LV brain natriuretic peptide mRNA as measured by qPCR. (C) LV atrial natriuretic peptide mRNA as measured by qPCR. *p≤0.05, **p≤0.01, ***p≤0.001, NS: non-significant.

Hypercholesterolemic rabbits showed an increase of LV mass indexed to body weight over time (p<0.001), while the normal diet rabbit group did not show significant changes (p = 0.387), resulting in a significant difference between the two diets (p<0.001; Fig 3A). Comparisons between groups at each time point indicate that this difference becomes significant at 14 weeks of hypercholesterolemic diet. Natriuretic peptide levels are known to be associated with LV filling pressures. In our study, hypercholesterolemic rabbits showed a significant increase of brain natriuretic peptide (*Bnp*) mRNA levels compared to that in the normal group (p = 0.006; Fig 3B). Cardiac mRNA level of atrial natriuretic peptide (*Anp*) was also increased but the difference did not reach significance (p = 0.151; Fig 3C).

### Hypercholesterolemic rabbits do not develop AVS in comparison to normal animals

**Fig 4:**
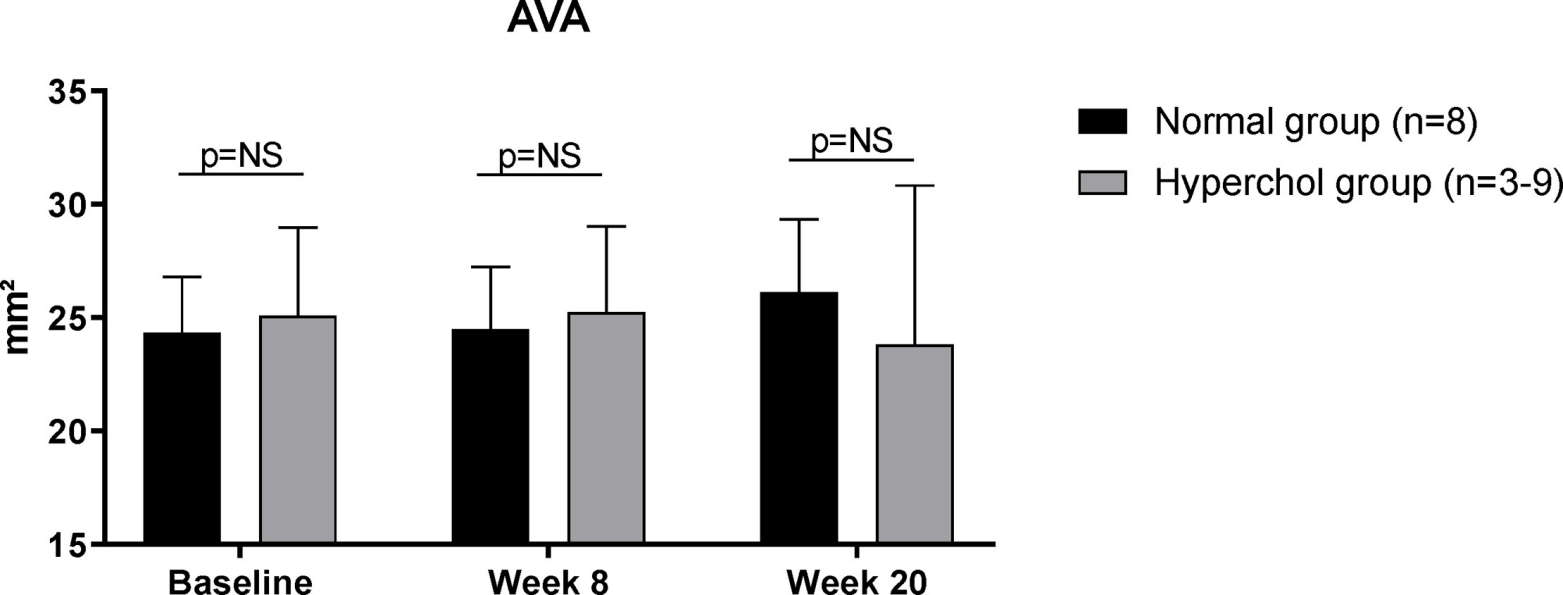
Aortic valve area (AVA) is not significantly different between groups in any time point of the study as measured by echocardiography. NS: non-significant.

Aortic valve area (AVA) was not significantly different between normal and hypercholesterolemic groups at week 20 of diet as shown in Fig 4 (baseline: 24.3±2.5 *vs* 25.1 ± 3.9 mm^2^; p = 0.638 and week 20: 26.1 ± 3.2 *vs* 23.8 ± 7.0 mm^2^; p = 0.122 in normal and hypercholesterolemic groups, respectively). Also, when compared to our previous study of hypercholesterolemic diet with vitamin D_2_ supplementation [10], our rabbits without vitamin D_2_ did not show any sign of aortic valve stenosis (S2 Fig).

### High cholesterol diet causes cardiac inflammation in rabbits

**Fig 5:**
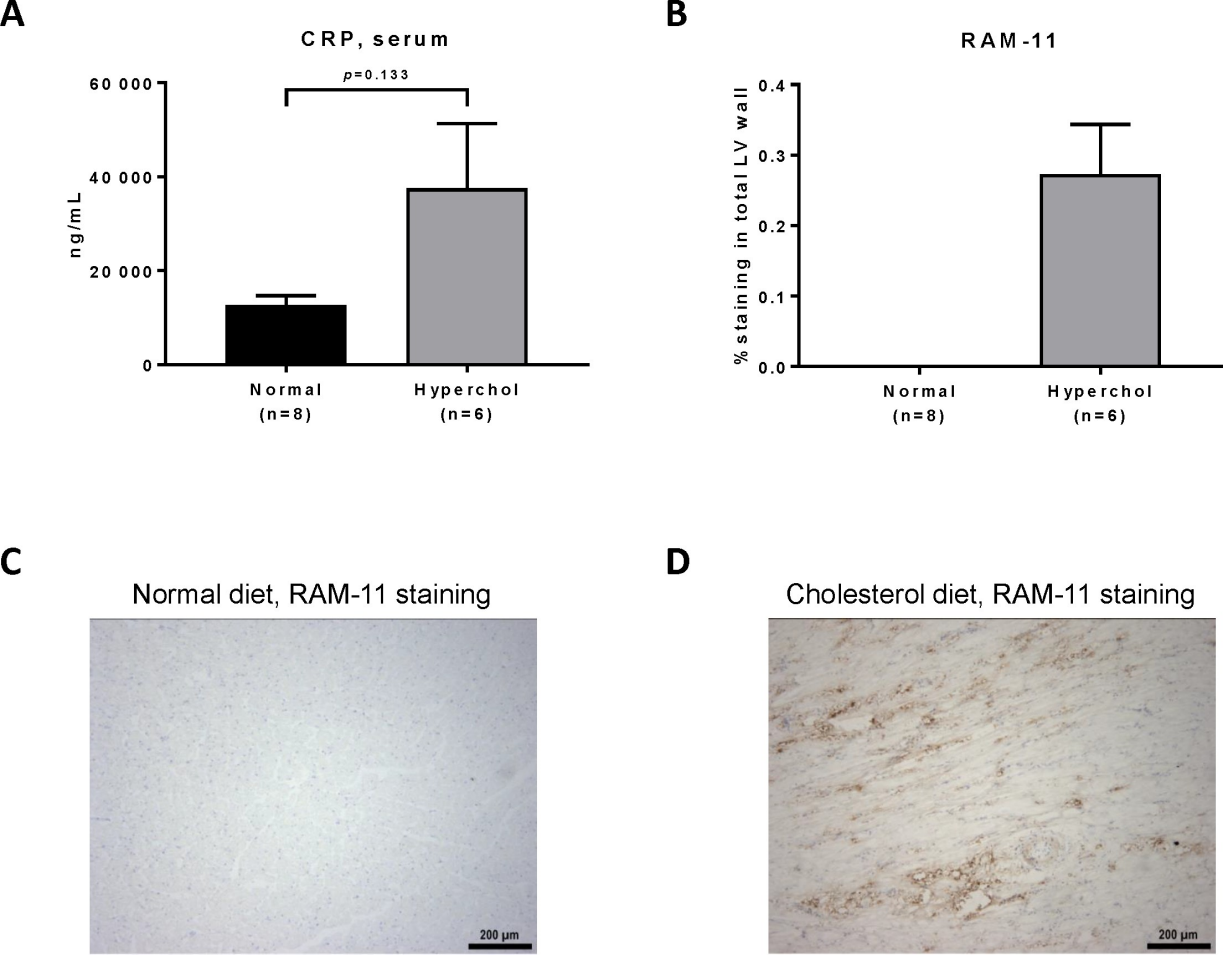
Inflammation markers in rabbits assessed by immunohistochemistry and biochemistry analysis. (A): C-reactive protein levels in rabbits’ serum quantified by ELISA at the end of the study. (B): Macrophage percentage in LV wall as revealed by RAM-11 antibody. (C): RAM-11 staining in normal rabbit, representative photo taken at 10x magnification. (D): RAM-11 staining in hypercholesterolemic rabbit, representative photo taken at 10x magnification.

**Fig 6:**
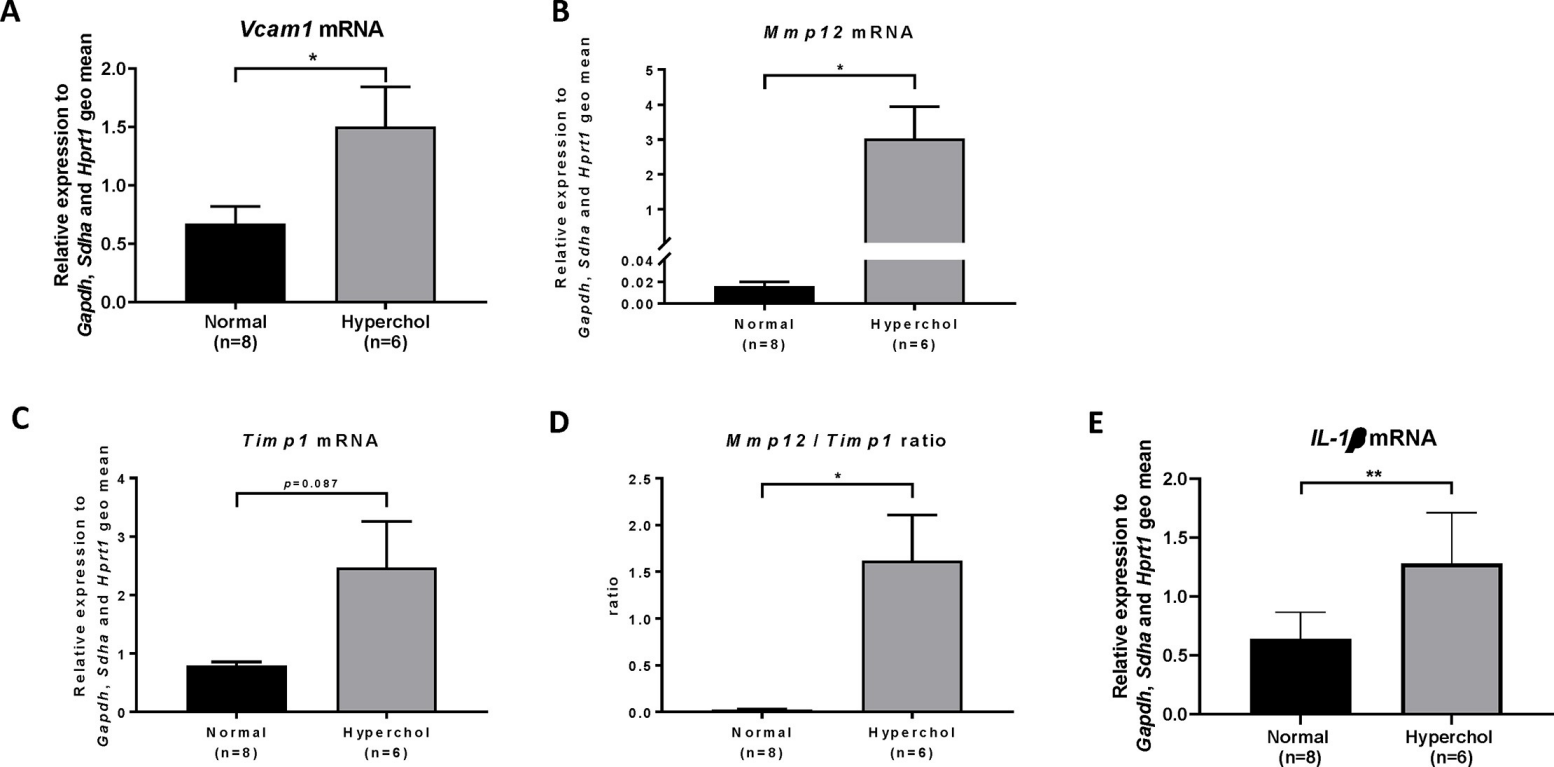
Inflammatory markers quantified by qPCR in LV homogenates after sacrifice. (A): *Vcam1* mRNA, (B): *Mmp12* mRNA, (C): *Timp1* mRNA, (D): *Mmp12*/*Timp1* mRNA ratio, (E) *Il1b* mRNA. *p≤0.05, **p≤0.01.

Serum CRP levels were numerically higher in hypercholesterolemic rabbits compared to the normal diet group (p = 0.133; Fig 5A). Hypercholesterolemic rabbits also presented macrophage infiltration as revealed by RAM-11 staining in LV sections (27.2 ± 7.1% of LV area; Fig 5B and 5D) whereas no staining could be detected in normal rabbits (Fig 5C, p<0.001 between groups). Quantitative PCR analysis showed an increase of cardiac mRNA levels of vascular cell adhesion molecule 1 (*Vcam1*, p = 0.030; Fig 6A), matrix metalloproteinase 12 (*Mmp12*, p = 0.023; Fig 6B), tissue inhibitor of metalloproteinases 1 (*Timp1*, p = 0.087; Fig 6C) the ratio of *Mmp12* to *Timp1* (p = 0.022; Fig 6D) and interleukin 1β (*Il1b*, p = 0.004; Fig 6E) in hypercholesterolemic rabbits compared to normal animals. Multiple correlations were found between inflammatory markers and echocardiographic parameters suggesting an important role of inflammation in our model (S3 Fig). Thus, cardiac inflammation clearly develops when rabbits are exposed to a high cholesterol diet.

### High cholesterol diet induces myocardial oxidative stress in rabbits

**Fig 7:**
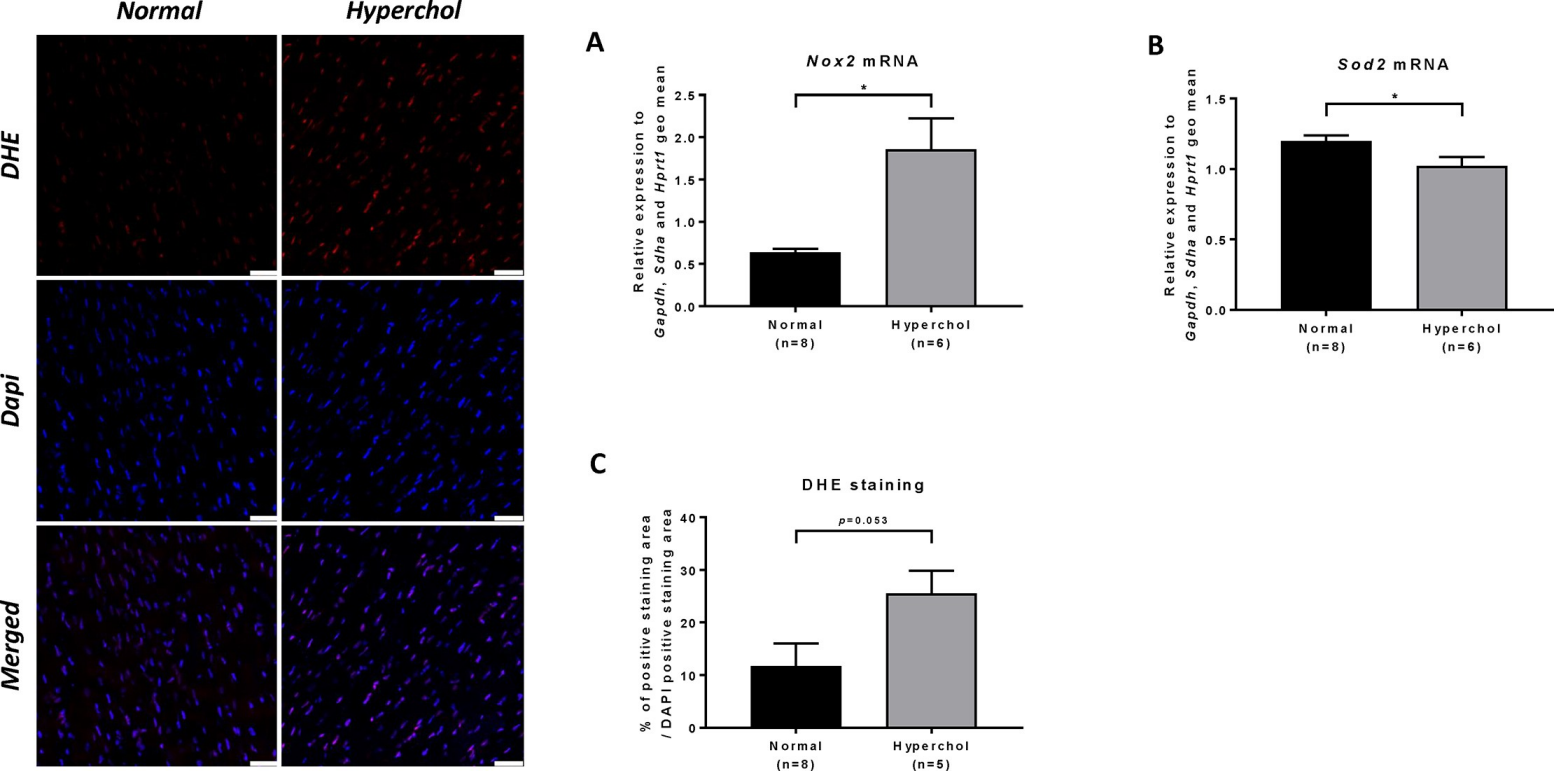
Oxidative stress status in rabbits. (A): *Nox2* mRNA levels in LV homogenates as quantified by qPCR, (B): *Sod2* mRNA levels in LV homogenates as quantified by qPCR, (C): % of DHE positive staining normalized to DAPI staining. *p≤0.05.

After an average period of 20 weeks of cholesterol-enriched diet, cardiac mRNA level for the NADPH oxidase enzyme complex subunit *Nox2* was significantly increased (p = 0.021; Fig 7A) whereas that for the anti-oxidant enzyme type 2 superoxide dismutase was reduced (*Sod2*, p = 0.031; Fig 7B) compared to the normal group. Also, reactive oxygen species (ROS) generation was quantified in LV sections by the DHE method. Compared to the normal group, DHE staining was increased in hypercholesterolemic rabbits (25.6 ± 4.3 *vs* 11.7 ± 4.3%, p = 0.053; Fig 7C).

### Hypercholesterolemic rabbits present an altered ratio of mRNAs for collagen subtypes

**Fig 8:**
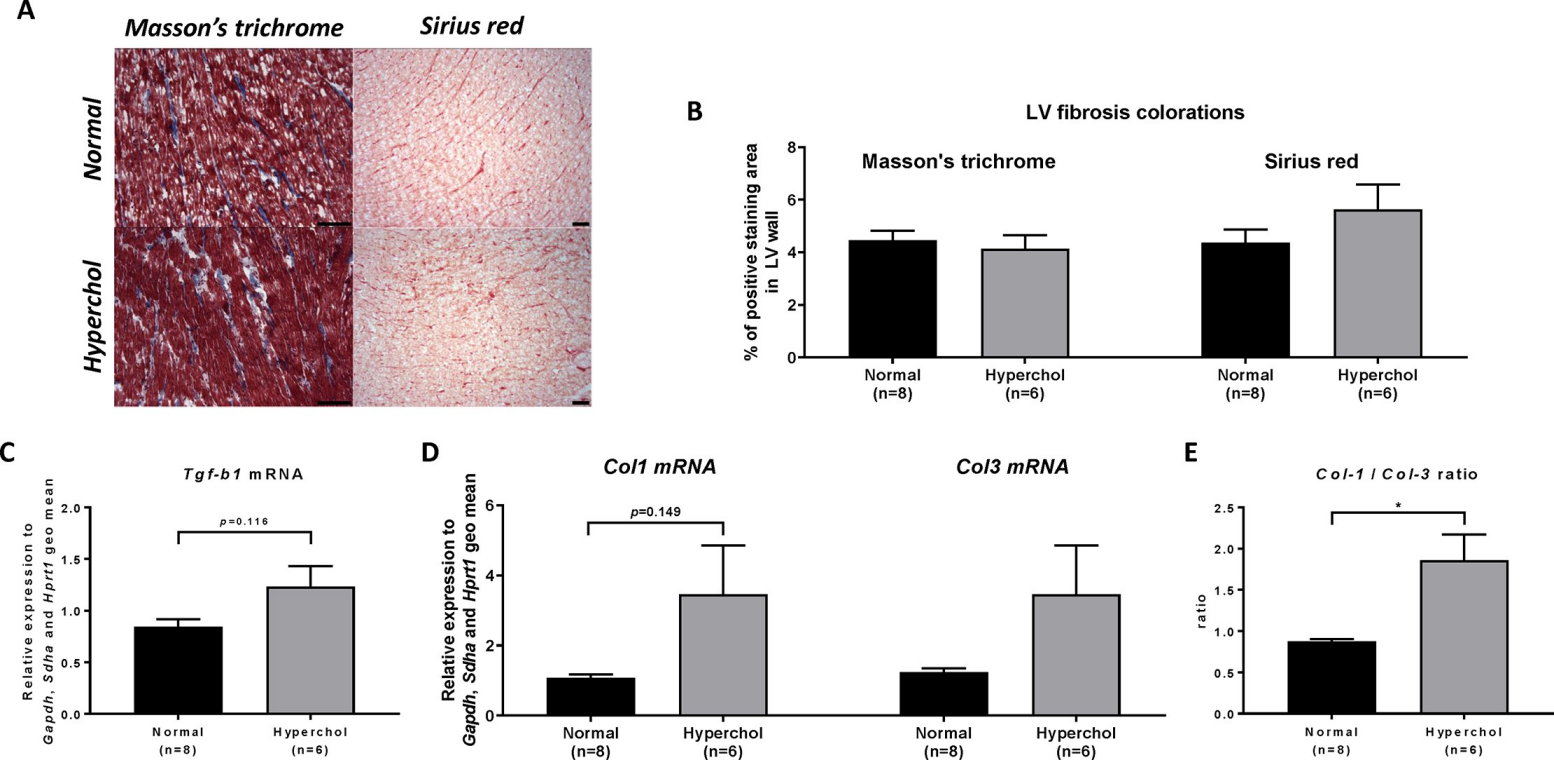
Cardiac fibrosis genes assessed by qPCR and histology marker of collagens fibers in LVs. (A) Masson’s trichrome and Sirius red representative photos from normal and hypercholesterolemic rabbits (B): Masson’s trichrome and Sirius red coloration quantified in LV sections at 10x magnification. (C): *Tgfb1* mRNA, (D): Collagen I mRNA, and Collagen III mRNA, (E): *Col1*/*Col3* mRNA ratio. *p≤0.05.

Cardiac fibrosis was assessed by Masson’s trichrome and Sirius red staining of LV sections and was not different between groups (p = 0.595 and p = 0.231 respectively; Fig 8A). At the molecular level, a trend towards increased cardiac mRNA levels was observed for *Tgfb1* (p = 0.116; Fig 8B) and *Col1* (p = 0.149; Fig 8C), but not for *Col3* (p = 0.463; Fig 8D), in rabbits fed with the high cholesterol diet compared to control animals. In contrast, the *Col1* to *Col3* collagen ratio was significantly increased in hypercholesterolemic rabbits in comparison to normal animals (p = 0.026; Fig 8E).

## Discussion

Diastolic dysfunction is a complex disease that involves different pathways [13]. The lack of understanding of the molecular mechanisms behind the progression of this disease is mainly due to poor suitable animal models of LVDD [14,15]. Hypercholesterolemia is one of the main risk factor of coronary artery disease but its role in LVDD has been underappreciated. A model of both systolic and diastolic dysfunction in rabbits exposed to a high cholesterol diet for 10 weeks was previously described [16]. A large animal model chronically exposed to three common comorbidities that associate with LVDD (*i*.*e*. hyperglycemia, hypercholesterolemia and hypertension) was also recently reported [17]. The present study aimed to develop a simple lipid-mediated LVDD rabbit model, without valvular dysfunction nor LV systolic dysfunction, using the tissue-Doppler parameter E/Em ratio as the primary endpoint.

Diastolic function encompasses all processes involved in LV filling and includes both active ones (energy-dependent), including myocardial relaxation, and passive characteristics such as loading conditions and myocardial compliance. Doppler echocardiography plays a central role in the evaluation of LV diastolic function [4]. In this study, we chose to evaluate serially the mitral E/Em ratio and to define LVDD when a value of this echocardiographic parameter was higher than the mean at baseline + 2SD. Most of our rabbits met this criterion after a 15 or 16-week period of hypercholesterolemic diet. LVDD was then also characterized by other echocardiographic parameters including increased mitral E-wave velocity, increased mitral E-wave deceleration rate (suggesting altered LV compliance), increased difference between A-wave and Ar-wave durations (indicating higher LV end diastolic pressure), and increased indexed left atrial diameter (reflecting the cumulative effects of increased LV filling pressures over time). Altogether, these data strongly support the presence of LVDD with preserved systolic function in our hypercholesterolemic rabbits.

Inflammation has been reported to be a key player in LVDD [18]. During the last few years, a few studies have supported the paradigm proposed by Paulus and Tschöpe [19,20], whereby systemic inflammation promotes coronary endothelial dysfunction [21], allowing myocardial leucocyte infiltration and inflammation. These pathological features have all been observed in the lipid-mediated rabbit model presented in this study. First, hypercholesterolemic rabbits presented a pro-inflammatory state as documented by elevated levels of circulating CRP and by the presence of macrophages observed on LV sections. The presence of inflammation was also supported by the increased expression in the myocardium of mRNA coding for *Il1b* and for *Vcam1*, an adhesion molecule responsible for the adhesion of leukocytes to the endothelium [22], suggesting endothelial dysfunction. Second, hypercholesterolemic rabbits showed increased myocardial oxidative stress as reflected by changes of mRNA levels of the pro-oxidant NADPH enzyme subunit (increased) and of the anti-oxidant enzyme Sod-2 (decreased). This was accompanied by higher levels of ROS generation as shown by increased DHE staining. It has been reported that oxidative depletion of NOS co-factor, tetrahydrobiopterin (BH_4_), reduces NO bioavailability and causes endothelial and diastolic dysfunction. Indeed, uncoupling of NOS with BH4 leads to production of superoxide (O_2_^-^) instead of NO [23]. This increased oxidative stress state indicates cardiac cell dysfunction.

Third, our hypercholesterolemic rabbits presented an increased *Col1* to *Col3* collagen mRNA ratio in comparison to normal animals on qPCR experiments, with a similar trend for *Tgfb1*. Our rabbits fed with a high cholesterol diet also showed increased mRNA levels of *Mmp12* and *Timp1*, suggesting enhanced cardiac extra-cellular matrix remodeling. As MMP12 targets elastin, and TIMP1 inhibits matrix proteolysis and therefore results in matrix accumulation, these changes might be responsible for the increased myocardial stiffness (*i*.*e*. decreased myocardial compliance) contributing to LVDD.

Finally, our rabbits exposed to high cholesterol diet developed signs of liver dysfunction (increased liver mass and enzyme levels). Among 50% of cirrhotic patients presents LVDD [24]. Liver dysfunction may be involved, at least partly, in the progression of LVDD in our model. This hepatic dysfunction is probably caused by the high cholesterol levels observed in this model.

## Conclusion

We characterized a simple and reliable lipid-mediated LVDD rabbit model which has been validated with robust echocardiography parameters. This model presented features classically observed in LVDD such as cardiac inflammatory state and increased oxidative stress, as well as preserved LV systolic function. This rabbit model may be used in future studies to test treatment strategies against LVDD and heart failure with preserved ejection fraction (HFpEF).

## Limitations

LVDD was characterized by echocardiography only. However, invasive hemodynamic parameters are still considered the gold standard in clinic for the detection of LVDD. We did not measure lung weights in our rabbits, which is an important indicator of clinically relevant fluid retention in this pathology. Finally, our hypercholesterolemic rabbits develop coronary artery disease with a high percentage of coronary obstruction. This may cause a concomitant ischemic heart disease with LVDD presence and could affect the interpretation of future studies to treat LVDD.

## Supporting information

S1 Fig Systolic parameters were stable between groups over time as assessed by echocardiography. EF: ejection fraction. FS: Fractional shortening.
(TIFF)

S2 Fig AVS parameters are less affected by high cholesterol diet only than by high cholesterol diet with presence of vitamin D_2_ (rabbits with vitamin D2 reported in our previous study [9]]). AVA: Aortic valve area, G_mean_: Mean gradient cross AV flow, G_peak_: Peak gradient cross AV flow. Calcification percentage and plaque area were assessed on left coronary leaflets and sinuses from rabbits fed with normal diet, high cholesterol diet alone and high cholesterol diet supplemented with vitamin D_2_ (the later being rabbits reported in our previous study [9]). Von kossa’s staining clearly indicates the essential role of vitamin D_2_ in the progression of AVS via calcification pathway. ***p<0.001.
(TIFF)

S3 Fig Multiple significant correlations between inflammatory markers (mRNA expression of *Il1b*, *Vcam1* and *Mmp12*) and representative LVDD echocardiography parameters (LADd, E/A ratio, lateral E/Em ratio et lateral Em/Am ratio, A-Ar_Left_ durations). Pearson correlations were performed when data was normally distributed and Spearman correlation when data was not normally distributed. A-Ar durations: mitral A-wave (active atrial filling) duration *minus* left pulmonary venous reversed atrial flow duration, Am: mitral annulus velocity during active atrial filling, E: peak velocity during early left ventricular filling, Em: mitral annulus velocity during early left ventricular filling, LADd: smallest left atrium dimension at end cardiac diastole.
(TIFF)

S4 Fig Left atrial (LA) dimension at end diastole (LADd) and at end systole (LADs) and heart rate (HR) echocardiographic parameters in normal and hypercholesterolemic rabbits. No significant change between groups was obtained for HR. However, LADd and LADs increased over time in hypercholesterolemic group when compared to normal group. *p≤0.05, **p≤0.01, ***p≤0.001. Statistical analyses were performed to assess parameters’ change over time in each group, comparison between the change over time between groups and the differences between groups at each time points.
(TIFF)

S1 Table Forward and reverse primers of housekeeping genes and genes of interest. (PDF)

S2 Table Body and organ weights in normal and high cholesterol diet groups at end of study. (PDF)

S3 Table Metabolic biomarkers in normal and high cholesterol diet groups at baseline and end of study. (PDF)

S4 Table Liver enzymes in normal and high cholesterol diet groups at baseline and end of study. (PDF)

S5 Table Coronary lumen and plaque area in normal and high cholesterol diet groups at end of study. (PDF)
