# Supplementary figures and images for: Cardiac inflammation and diastolic dysfunction in hypercholesterolemic rabbits
Source: PLoS One. 2019 Aug 8;14(8):e0220707. doi: 10.1371/journal.pone.0220707 (PMC6687122; doi:10.1371/journal.pone.0220707)

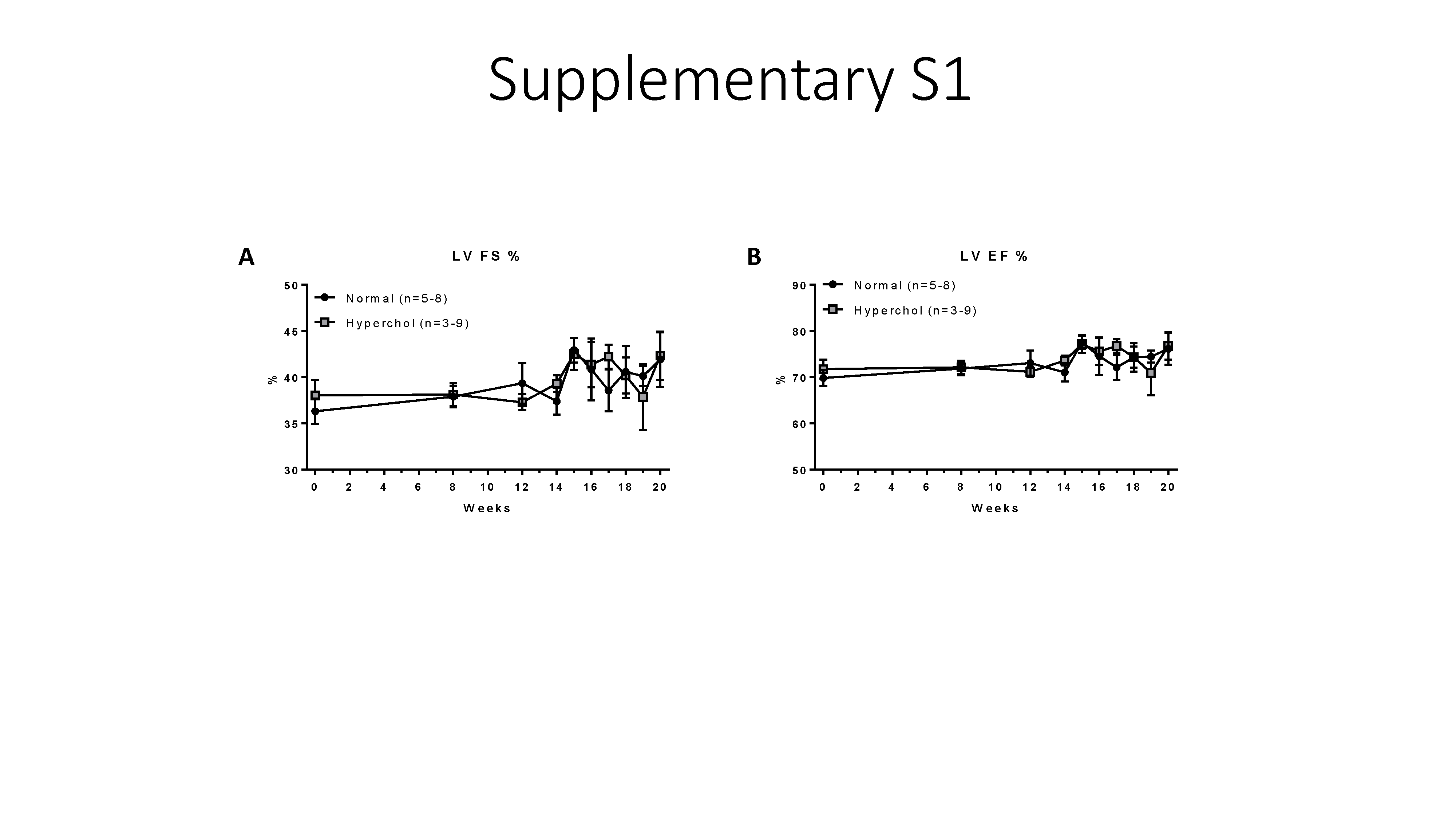

Supplement: S1 Fig — EF: ejection fraction. FS: Fractional shortening. (TIFF) [file pone.0220707.s001.tiff]

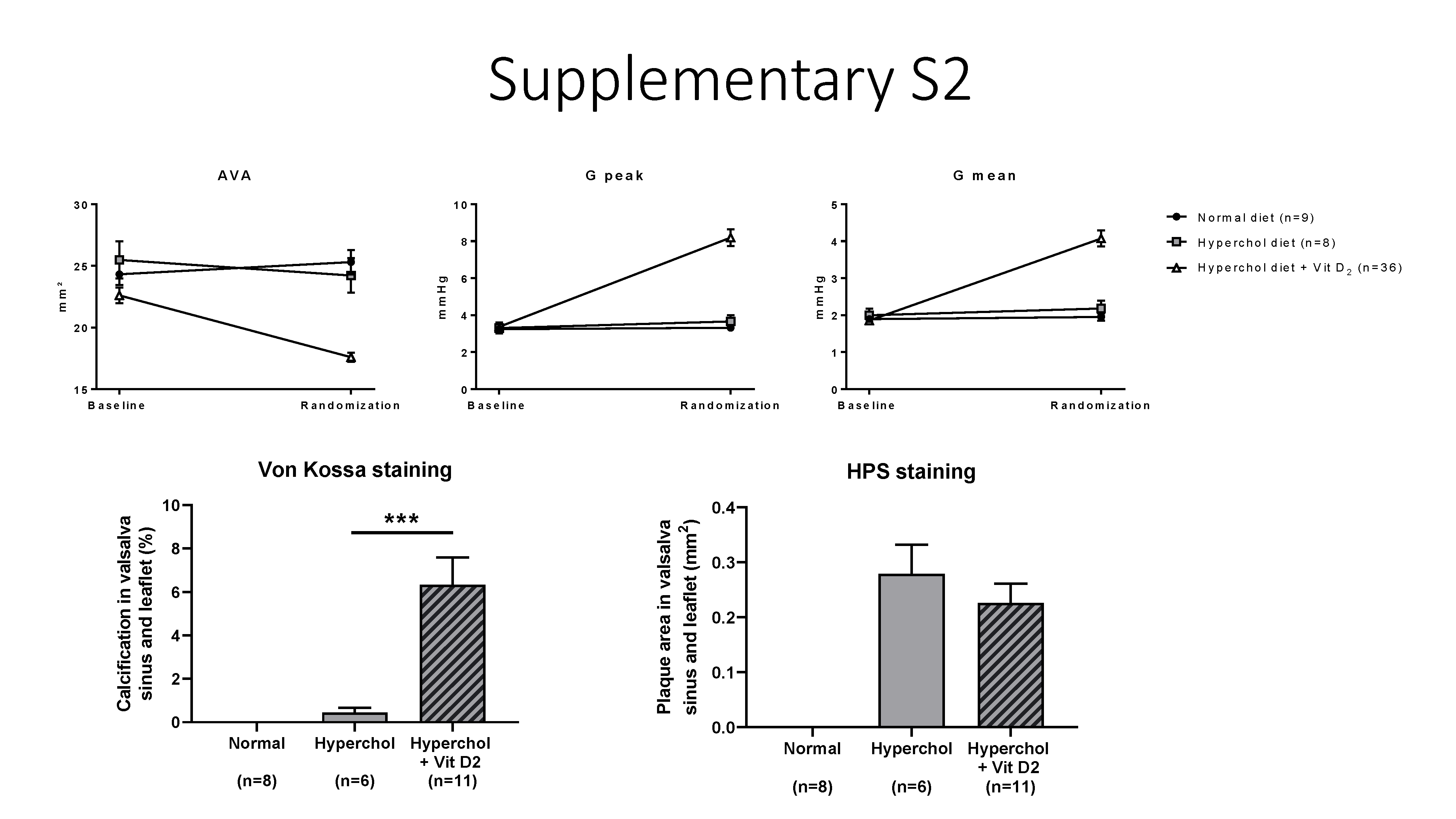

Supplement: S2 Fig — AVA: Aortic valve area, Gmean: Mean gradient cross AV flow, Gpeak: Peak gradient cross AV flow. Calcification percentage and plaque area were assessed on left coronary leaflets and sinuses from rabbits fed with normal diet, high cholesterol diet alone and high cholesterol diet supplemented with vitamin D2 (the later being rabbits reported in our previous study [9]). Von kossa’s staining clearly indicates the essential role of vitamin D2 in the progression of AVS via calcification pathway. ***p<0.001. (TIFF) [file pone.0220707.s002.tiff]

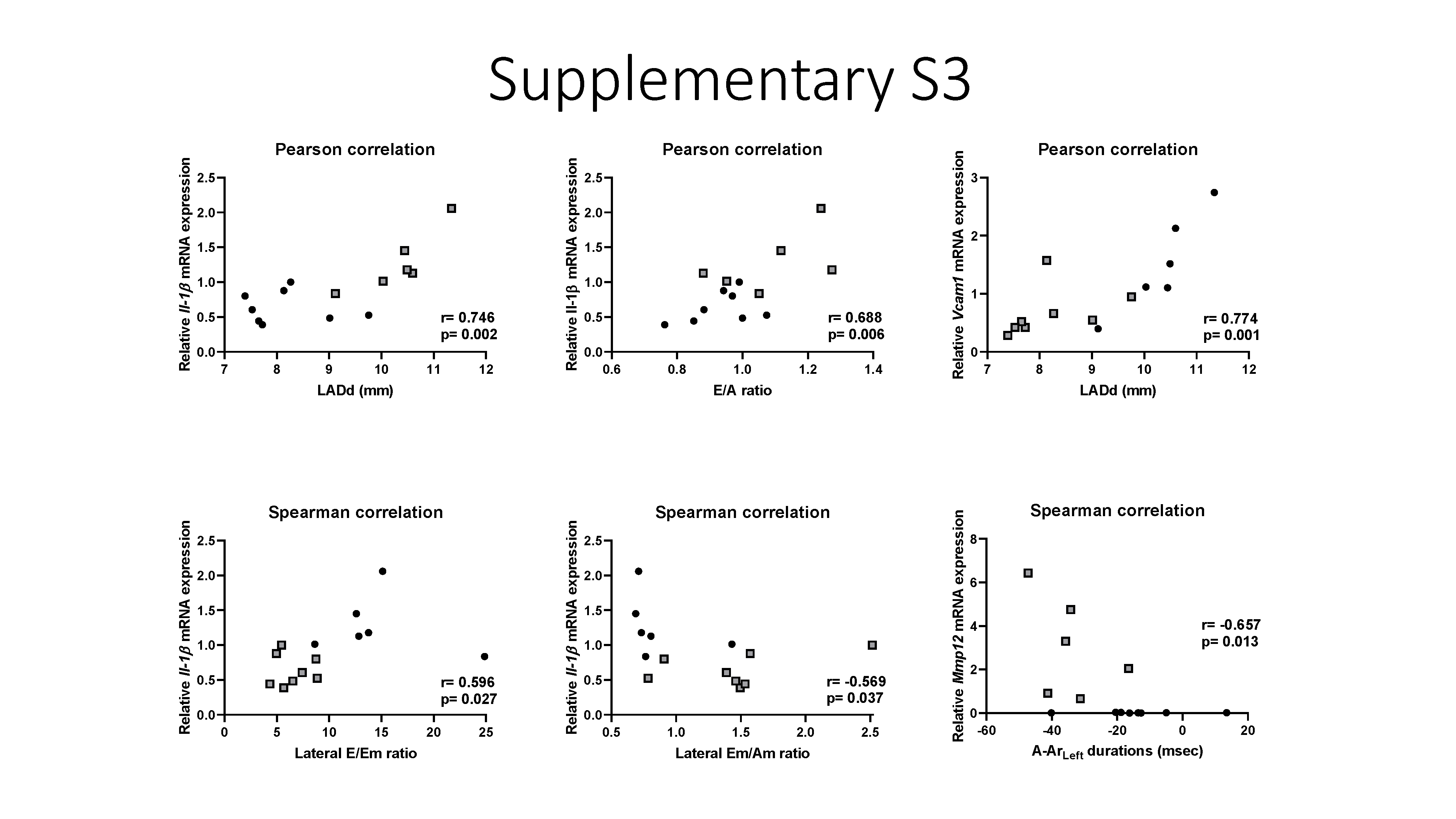

Supplement: S3 Fig — Pearson correlations were performed when data was normally distributed and Spearman correlation when data was not normally distributed. A-Ar durations: mitral A-wave (active atrial filling) duration minus left pulmonary venous reversed atrial flow duration, Am: mitral annulus velocity during active atrial filling, E: peak velocity during early left ventricular filling, Em: mitral annulus velocity during early left ventricular filling, LADd: smallest left atrium dimension at end cardiac diastole. (TIFF) [file pone.0220707.s003.tiff]

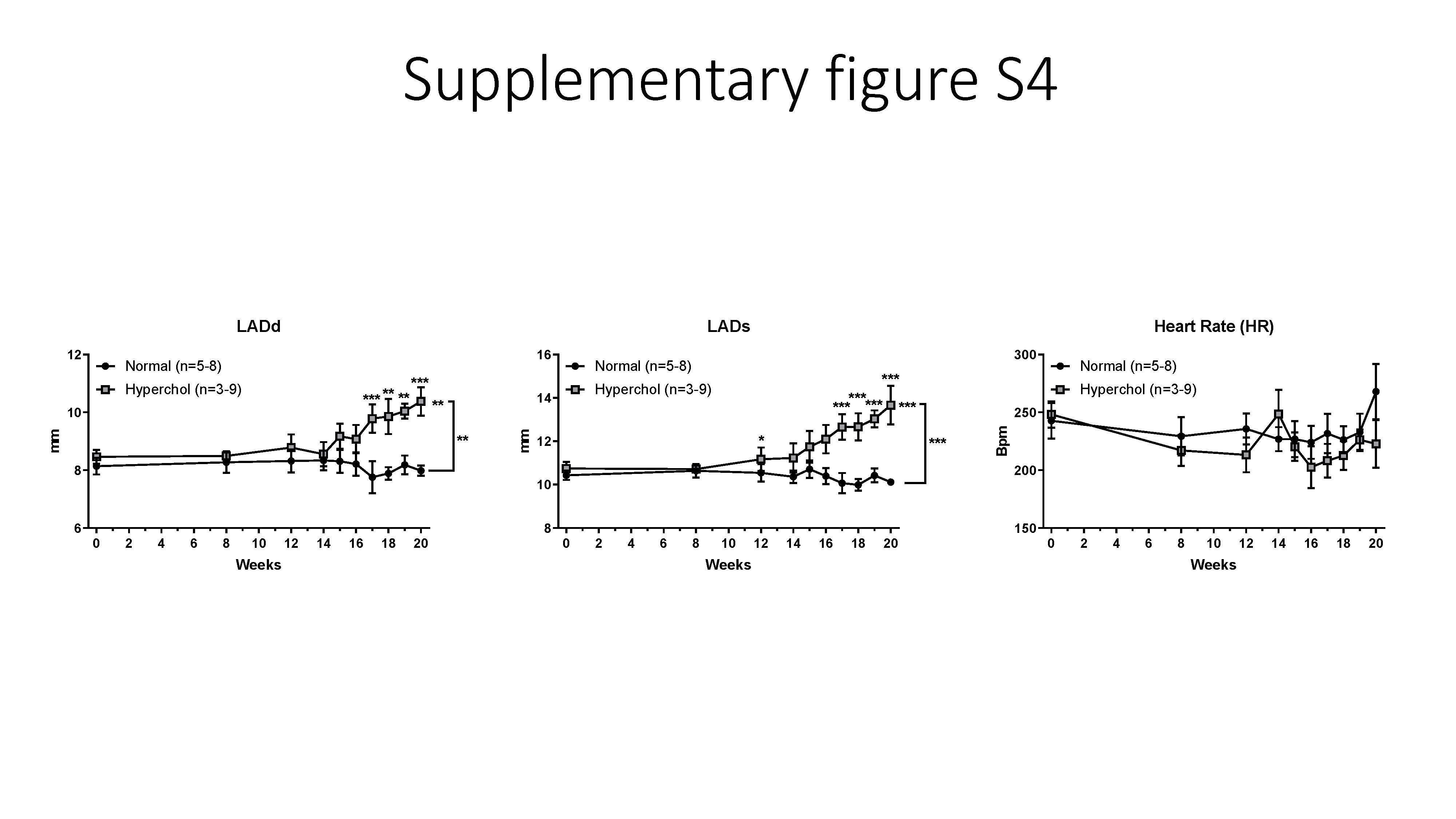

Supplement: S4 Fig — No significant change between groups was obtained for HR. However, LADd and LADs increased over time in hypercholesterolemic group when compared to normal group. *p≤0.05, **p≤0.01, ***p≤0.001. Statistical analyses were performed to assess parameters’ change over time in each group, comparison between the change over time between groups and the differences between groups at each time points. (TIFF) [file pone.0220707.s004.tiff]
