# Supplementary material for: Cardiac inflammation and diastolic dysfunction in hypercholesterolemic rabbits
Source: PLoS One. 2019 Aug 8;14(8):e0220707. doi: 10.1371/journal.pone.0220707 (PMC6687122; doi:10.1371/journal.pone.0220707)
Supplement: S1 Table — (PDF) [file pone.0220707.s005.pdf]

Supplementary Table S1

Supplementary Table S1: Forward and reverse primers of housekeeping genes and genes of interest

Gene Symbol	Accession number	Forward primer (5'-3')	Reverse primer (5'-3')
Gapdh	NM_001082253	TGGTGAAGGTCGGAGTGAAC	ATGTAGTGGAGGTCAATGAATGG
Hprt1	NM_001105671	CCTTGGTCAAGCAGTATAATC	GGGCATATCCTACAACAAAC
Sdha	XM_002723194	GGACCAGGACGCCATCCACTAC	TCCACCGAACGCACGCTGATAG
Bnp	XM_008275384	CCTGTGCCCCTGGATGAG	CCGAAGCAGCCTGAGTCC
Anp	NM_001082262	GTACAACGCCATGTCCAAC	CTTCATCACTCTGCTCACTTAG
Vcam1	NM_001082152	GAACTTCTCGTGCTCTATTGTG	TGTGCTTCTACCAGACTGTATG
Mmp12	NM_001082771	GGCAACTGGACACATCTAC	GGCATACTCAACATCCTCAC
Timp1	NM_001082232	CTCATCGCTGGACAACTG	GCGTAGGTCTTGGTGAAG
Nox2	NM_001082100	AATGCTTGTGGCTGTGATAAG	TACCAGACTGACTTGAGGATG
Sod2	XM_008263745	CAAACCTGAGCCCTAACG	TGTTCCCACACATCAATCC
Tgf-β1	XM_008249704	AGATAATGTCCTCCAAGTG	GAACTGTGTAGATGTTGAG
Col 1	XM_017348831	AGCCAGCAGATTGAGAAC	TCGCAGAAGACCTTGATG
Col 3	XM_002712333	CCTGAAGCCCCAGCAGAAAATTG	AGTTGGTCACTTGTACTGGTTGAC
Il-1β	NM_001082201	AATTACATGAAGAGCTGCTT	CACAACGACTGACAAGAC
