# Supplementary material for: Cardiac inflammation and diastolic dysfunction in hypercholesterolemic rabbits
Source: PLoS One. 2019 Aug 8;14(8):e0220707. doi: 10.1371/journal.pone.0220707 (PMC6687122; doi:10.1371/journal.pone.0220707)
Supplement: S2 Table — (PDF) [file pone.0220707.s006.pdf]

Supplementary Table S2

Supplementary Table S2: Body and organ weights in normal and high cholesterol diet groups at end of study

	Normal diet group	High cholesterol diet group	*p*-value
Body weight (BW) kg	4.04 ± 0.42	3.63 ± 0.38	0.0696
Heart weight/BW (g/kg)	1.96 ± 0.17	2.22 ± 0.38	0.1151
Left ventricle weight/BW (g/Kg)	1.42 ± 0.13	1.75 ± 0.22	0.0001
Liver weight/BW (g/kg)	25.6 ± 4.5	35.3 ± 4.2	0.0014
Spleen weight/BW (g/kg)	0.39 ± 0.12	1.31 ± 1.02	0.0778
Right kidney weight/BW (g/kg)	2.08 ± 0.14	2.79 ± 0.36	0.0035
Left kidney weight/BW (g/kg)	2.11 ± 0.14	2.78 ± 0.43	0.0109

Results are expressed as mean ± SEM
