# Supplementary material for: Cardiac inflammation and diastolic dysfunction in hypercholesterolemic rabbits
Source: PLoS One. 2019 Aug 8;14(8):e0220707. doi: 10.1371/journal.pone.0220707 (PMC6687122; doi:10.1371/journal.pone.0220707)
Supplement: S3 Table — (PDF) [file pone.0220707.s007.pdf]

Supplementary Table S3

Supplementary Table S3: Metabolic biomarkers in normal and high cholesterol diet groups at baseline and end of study

Metabolic biomarker	Time point	Normal diet group	High cholesterol diet group	*p*-value
Glucose (mmol/L)	Baseline	8.31 ± 0.49	7.81 ± 0.19	0.3780
	End of Study	8.18 ± 0.32	6.83 ± 0.21	0.0048
Uric Acid (mmol/L)	Baseline	15.2 ± 1. 5	13.2 ± 0.6	0.2492
	End of Study	14.9 ± 1.5	70.5 ± 24.9	0.0757

Results are expressed as mean ± SEM
