# Supplementary material for: Cardiac inflammation and diastolic dysfunction in hypercholesterolemic rabbits
Source: PLoS One. 2019 Aug 8;14(8):e0220707. doi: 10.1371/journal.pone.0220707 (PMC6687122; doi:10.1371/journal.pone.0220707)
Supplement: S4 Table — (PDF) [file pone.0220707.s008.pdf]

Supplementary Table S4

Supplementary Table S4: Liver enzymes in normal and high cholesterol diet groups at baseline and end of study

Liver enzymes	Time point	Normal diet group	High cholesterol diet group	*p*-value
AST (U/L)	Baseline	36.4 ± 5.1	34.5 ± 2.7	0.7508
	End of Study	16.0 ± 1.4	41.9 ± 25.0	0.3582
ALT (U/L)	Baseline	49.0 ± 6.1	45.8 ± 7.1	0.7407
	End of Study	29.5 ± 2.8	46.1 ± 3.1	0.0048

Results are expressed as mean ± SEM
