# Supplementary material for: Cardiac inflammation and diastolic dysfunction in hypercholesterolemic rabbits
Source: PLoS One. 2019 Aug 8;14(8):e0220707. doi: 10.1371/journal.pone.0220707 (PMC6687122; doi:10.1371/journal.pone.0220707)
Supplement: S5 Table — (PDF) [file pone.0220707.s009.pdf]

Supplementary Table S5

Supplementary Table S5: Coronary lumen and plaque area in normal and high cholesterol diet groups at end of study

	Normal diet group	High cholesterol diet group	*p*-value
Coronary lumen area (μm^2)	18. 9 ± 3.7	3.8 ± 1.8	0.0055
Coronary plaque area (μm^2)	0.0 ± 0.0	25.7 ± 5.8	-
Coronary obstruction (%)	0.0 ± 0.0	85.7 ± 3.6	-

Results are expressed as mean ± SEM
